# The influence of RFI classification and cow age on body weight and body condition change, supplement intake, and grazing behavior of beef cattle winter grazing mixed-grass rangelands

**DOI:** 10.1093/tas/txaa100

**Published:** 2020-12-22

**Authors:** Cory T Parsons, Julia M Dafoe, Samuel A Wyffels, Megan Van Emon, Timothy DelCurto, Darrin L Boss

**Affiliations:** 1 Northern Agricultural Research Center, Montana State University, Havre, MT; 2 Department of Animal and Range Sciences, Montana State University, Bozeman, MT

## INTRODUCTION

Providing adequate nutrition for animals is the greatest operating cost for cow–calf producers where supplemental feed can account for 65% of the annual expenses to maintain a cow–calf operation (Arthur et al., 2004; Van der Westhuizen et al., 2004; Meyer et al., 2008). In addition, the USDA Economic Research Service estimated that feed-associated costs comprised greater than 55% of all nonfixed costs of U.S. cow–calf operations (USDA-ERS, 2005). Traditionally, selection pressure has been placed on production traits associated with increasing outputs, which can also result in increased inputs to meet animal production potential. Since feed costs constitute the greatest proportion of total inputs, selection pressure for efficient animals that have lower feed intake but maintain production could have a great impact on cow–calf profitability (Meyer et al., 2008). It is estimated that two-thirds of feed energy is required for body maintenance (Ferrell and Jenkins 1984, 1988; Montaño-Bermudez et al. 1990), and substantial animal-to-animal variations, independent of body size and growth, exists in maintenance requirements of cattle (Arthur et al., 2001; Nkrumah et al., 2006; Crowley et al., 2010). Thus, improving feed efficiency through genetic selection holds significant opportunity for the beef industry.

Residual feed intake (RFI) is currently being used as a selection tool for purchasing and retaining heifers and for selecting bulls and semen. However, the use and relevance of RFI as a selection tool for the cow–calf industry in the western United States needs additional research. Little if any research supports selection for beef cows that fit western rangeland beef cattle systems using RFI values obtained in post-weaning studies. Most RFI studies have included energy-dense diets and rations focusing on feedlot performance (Lawrence et al., 2014). Research pertaining to RFI in cattle offered forage-based diets is limited (Arthur et al., 2005), with even fewer data available that relate to beef cows (Basarab et al., 2007; Meyer et al., 2008; Sprinkle et al., 2020). As a result, more research is needed to evaluate the utility of RFI estimates on the lifetime production of beef cattle in extensive forage base systems (Manafiazar et al., 2015; Sprinkle et al., 2020). Therefore, the objectives of this study are to evaluate the influence of cow RFI classification and cow age on weight and body condition change, as well as supplement intake and grazing behavior of winter grazing beef cattle. We hypothesized that there is no difference between cow RFI classification and cow age on production, supplement intake, nor grazing behavior.

## MATERIALS AND METHODS

The use of animals in this study was approved by the Institutional Animal Care and Use Committee of Montana State University AACUC #2018-AA12.

A 2-yr winter grazing study was conducted with nonlactating commercial Angus cows to evaluate the influence of RFI classification and cow age on supplement intake behavior, beef cattle performance, and grazing behavior. This study was conducted at the Montana State University Northern Agriculture Research Center’s Thackeray Ranch (48°21″N 109°30′W), located 21 km south of Havre, MT. The local climate is characterized as semi-arid steppe with an average annual precipitation of 410 mm. Vegetation is dominated by Kentucky bluegrass (*Poa pratensis* L.), bluebunch wheatgrass (*Pseudoroegneria spicata* [Pursh] A. Love), and rough fescue (*Festuca scabrella* Torr.; Wyffels et al., 2018).

A commercial herd of 205 (year 1) and 203 (year 2) bred Angus cows ranging in age from 1 to 9 yr old grazed two adjacent rangeland pastures, (Arches, 257 ha, ~1.1 ha ∙ AUM^−1^; and Anderson, 329 ha, ~1.5 ha ∙ AUM^−1^) from mid-October to early-January each year. All cows went through an RFI Growsafe trial (GrowSafe DAQ 4000E; GrowSafe System Ltd., Airdrie, AG, Canada) post-weaning (9 to 11 mo of age at the time of the trial) and were classified as either low (<0.50 SD from mean), average (± 0.50 SD from mean), or high (>0.50 SD from the mean) RFI within contemporary group. Cows were also grouped into six age classes (1, 2, 3, 4, 5 to 7, and ≥8 yr old) to evaluate the effects of RFI, and age on average daily individual supplement intake (g ∙ kg body weight^−1^ ∙ d^−1^), coefficient of variation (CV) of supplement intake (%), intake rate (g ∙ min^−1^), as well as changes in body weight (kg) and condition. Additionally, each year, cows were stratified by age (2, 5, and 8 yr olds) and RFI (high, low) and within strata, randomly assigned to wear 1 of 30 Lotek 3300LR GPS collars (Lotek Engineering, Newmarket, ON, Canada; five collars per RFI class within age class; Parsons et al., 2019).

All cows were provided free-choice access to a 28.7% crude protein (CP; year 1) and 30% CP (year 2) self-fed canola meal**-**based supplement with 23% salt to limit intake (Bovibox HM in year 1 and Bovibox in year 2; Table 1). The target daily-recommended intake range was 0.45 to 0.91 kg ∙ cow^−1^ ∙ d^−1^. Supplement was provided in a SmartFeed Pro self-feeder system to measure individual animal supplement intake and behavior. Supplement intake was measured during the last 45 d of grazing each year. Vegetation production was estimated by clipping 10 randomly located plots in each pasture pre-grazing using a 0.25 m^2^ plot frame. Samples were placed in a forced air oven at 55 °C for 72 h and then weighed and recorded to calculate dry matter production (kg ∙ ha^−1^; Table 2). Vegetation samples from each plot were ground to pass a 1-mm screen in a Wiley mill and sent to a commercial laboratory for nutrient analysis (Dairy One, Ithaca, NY).

**Table 1.** Guaranteed analysis of BoviBox protein block supplements

Guaranteed analysis	BoviBox HM (year 1)	BoviBox (year 2)
Crude protein	28.7% min	30% min
Crude fat	1.45% min	1.5% min
Crude fiber	5.0% max	5.0% max
Calcium	1.3% min	1.3% min
	1.8% max	1.8% max
Phosphorus	0.7% min	0.7% min
Salt	23% min	23% min
	26% max	
Potassium	1.5% min	1.5% min
Magnesium	2.5% min	1.0% min
Manganese	856 ppm	880 ppm
Zinc	1,074 ppm	1,100 ppm
Copper	213 ppm	220 ppm
Copper (from chelate)	108 ppm	110 ppm
Cobalt	15 ppm	16 ppm
Iodine	26 ppm	25 ppm
Selenium	3.3 ppm min	3.3 ppm min
	3.6 ppm max	3.6 ppm max
Selenium teast	—	1.7 ppm
Vitamin A	12,000 IU/lb	40,800 IU/lb
Vitamin D	4,000 IU/lb	4,500 IU/lb
Vitamin E	25 IU/lb	50 IU/lb
NPN not more than	9.70%	9.90%

**Table 2.** Average annual grass production (kg/ha), crude protein (CP %), acid detergent fiber (ADF %), neutral detergent fiber (NDF %), and total digestible nutrients (TDN %) of the experimental pastures for the 2 yr of grazing (2018–2019 and 2019–2020) at the Northern Agricultural Research Center Thackeray Ranch, Havre, MT

	Forage production (kg/ha)	CP (%)	ADF (%)	NDF (%)	TDN (%)
Year 1					
Arches	1901	7.8	41.0	62.9	56.0
Anderson	1790	5.4	41.9	63.2	56.0
Year 2					
Arches	1985	5.4	45.0	67.2	55.0
Anderson	1456	5.4	39.9	66.9	55.0

Supplement intake variables were analyzed using ANOVA with a generalized linear mixed model including RFI classification, age class, year, and the interaction of RFI, age class, and year as fixed effects, and individual cow as the random effect. Individual animal was considered the experimental unit, and an alpha ≤ 0.05 was considered significant. Orthogonal polynomial contrasts were used to determine linear and quadratic effects for each analysis. Means were separated using the Tukey method when *P* < 0.05. Tendencies were reported when significance was *P* ≤ 0.10. All statistical analyses were performed in R (R Core Team, 2017).

## RESULTS

**Figure 1.**
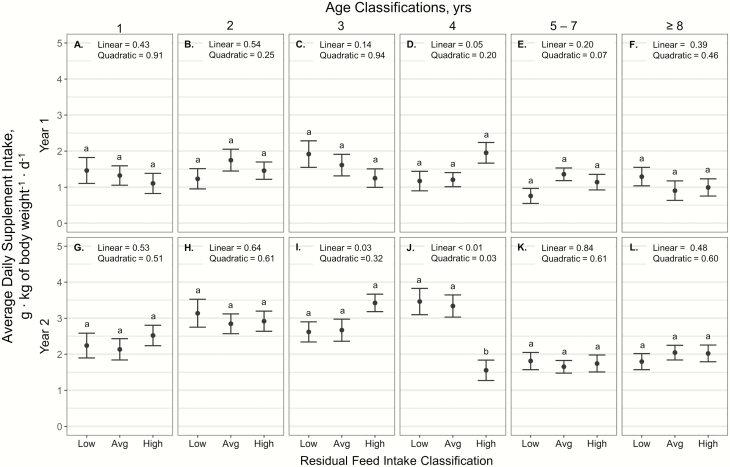
Influence of RFI classification within cow age classification and year (A–L) on average daily supplement intake (expressed as g ∙ kg^−1^ of body wt ∙ d^−1^ ± SE) by cattle grazing dormant mixed grass prairie in 2018–2019 and 2019–2020 at the MSU Northern Ag Research Center’s Thackeray Ranch, Havre, MT.

**Figure 2.**
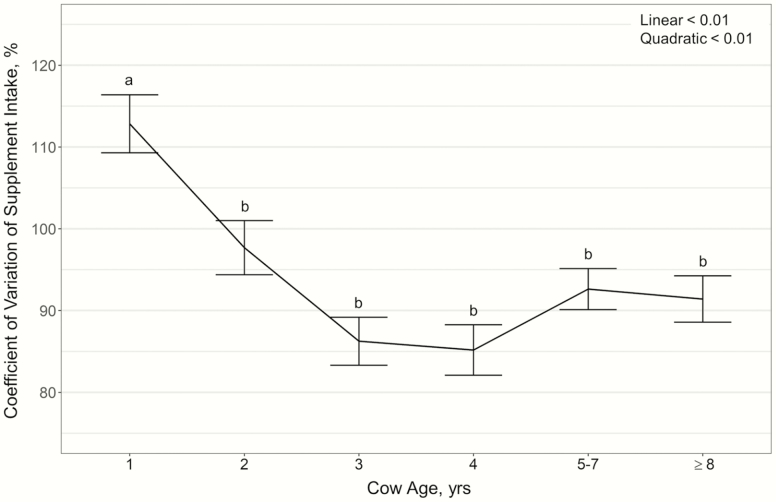
Influence of cow age on coefficient of variation of supplement intake (expressed as % ± SE) by cattle grazing dormant northern mixed grass prairie in 2018–2019 and 2019–2020 at the MSU Northern Ag Research Center’s Thackeray Ranch, Havre, MT.

Average daily supplement intake expressed as g ∙ kg body weight^−1^ ∙ d^−1^ displayed an RFI × age × year interaction (*P* < 0.01). In year 1, there was no effect of RFI classification within age group on supplement intake (*P* ≥ 0.07). In year 2, RFI class had a quadratic effect on supplement intake of 4-yr-old cattle (*P* = 0.03; Figure 1J), where high RFI cattle consumed less supplement per kg body weight than low and average RFI cattle (*P* < 0.01). Cow age displayed a quadratic effect on variation in supplement intake (% CV; *P* < 0.01; Figure 2). However, this effect was limited to 1-yr-old cattle having a larger CV of supplement intake than 2-, 3-, 4-, 5- to 7-, and ≥8-yr-old cows. Supplement intake rate differed by year (*P* < 0.01), where cows in year 1 consumed less supplement per minute than cows in year 2 (29.9 ± 1.81 and 91.8 ± 1.87 g ∙ min^−1^, respectively; *P* < 0.01). There was no effect of cow age or RFI classification observed on supplement intake rate (*P* = 0.99).

**Figure 3.**
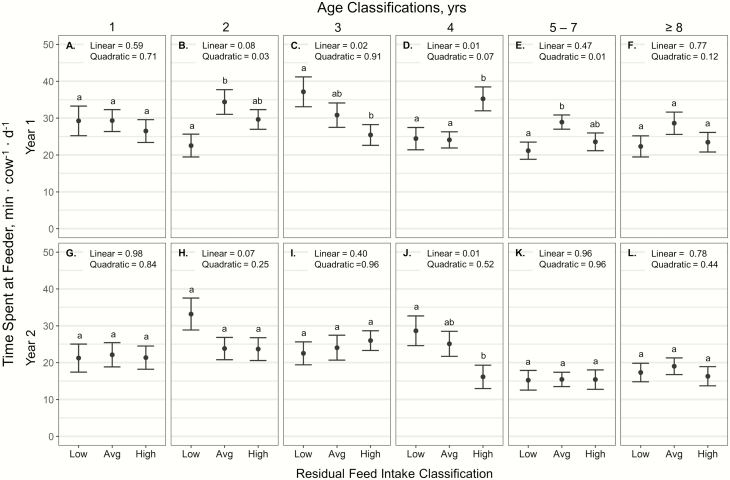
Influence of RFI classification within cow age classification and year (A–L) on average daily time spent at supplement feeder (expressed as min ∙ cow^−1^ ∙ d^−1^ ± SE) by cattle grazing dormant northern mixed grass prairie in 2018–2019 and 2019–2020 at the MSU Northern Ag Research Center’s Thackeray Ranch, Havre, MT, means within rows lacking common superscript differ (*P* < 0.05).

Daily time spent at the supplement feeders exhibited an RFI × age × year interaction (*P* < 0.01; Figure 3). During year 1, 3-yr-old cattle exhibited a negative linear response of RFI on time spent at the supplement feeders (*P* = 0.02; Figure 3C); however, 4-yr-old cattle had a positive linear response of RFI on time spent at the feeders (*P* = 0.01; Figure 3D). Additionally, RFI exhibited a quadratic effect on time spent at the supplement feeders for 2- and 5- to 7-yr old cattle in year 1 (*P* < 0.03; Figure 3B and E), where average RFI cattle spent more time at the feeder than low RFI cattle (*P* = 0.03). During year 2, 4 yr olds exhibited a negative linear response of RFI on time spent at supplement feeders (*P* = 0.01; Figure 3J).

Distance traveled and time spent grazing per day were neither effected by RFI classification nor year (*P* ≥ 0.19). However, cow age did display a tendency for a negative linear effect on distance traveled per day (*P* = 0.07), where 2 yr olds traveled 3.01 ± 0.06 km ∙ d^−1^, 5 yr olds traveled 2.73 ± 0.06 km ∙ d^−1^ and 8 yr olds traveled 2.49 km/d ± 0.06 km ∙ d^−1^.

Change in body condition score exhibited an RFI × age interaction (*P* = 0.05); however, no differences were observed among RFI classes when analyzed within age groups (*P* ≥ 0.27). There was a tendency for an age × year interaction for change in body weight (*P* = 0.06; Table 3), where in year 1, 1 yr old gained less than 2-, 3-, 4-, and ≥8 yr olds (*P* = 0.05), and in year 2, 5 to 7 yr olds lost more weight than ≥8 yr olds (*P* = 0.04). Overall, cows in year 1 gained an average of 26.3 ± 1.96 kg, whereas cows in year 2 lost an average of 19.2 ± 1.96 kg (*P* < 0.01).

**Table 3.** Average body weight and body condition score pretrial and changes to body weight and body condition score post-trial on six age classes of cattle (± SE) across a 2-yr grazing trial (2018–2019 and 2019–2020) at the Northern Agricultural Research Center Thackeray Ranch, Havre, MT

	Age class					
	1	2	3	4	5 to 7	≥8
Initial body weight, kg						
Year 1	489.6 ± 5.30	495.3 ± 7.60	565.4 ± 11.03	597.0 ± 8.88	617.2 ± 8.72	610.6 ± 9.28
Year 2	467.5 ± 7.55	508.4 ± 7.05	561.0 ± 9.19	616.7 ± 12.41	637.5 ± 6.75	624.6 ± 8.27
Initial body condition						
Year 1	5.76 ± 0.04	5.24 ± 0.08	5.52 ± 0.11	5.59 ± 0.09	5.56 ± 0.08	5.59 ± 0.07
Year 2	5.75 ± 0.05	5.21 ± 0.05	5.16 ± 0.11	5.47 ± 0.09	5.52 ± 0.06	5.41 ± 0.07
Δ Body weight, kg						
Year 1	9.25 ± 4.44^a^	29.35 ± 4.80^b^	32.65 ± 5.25^b^	28.84 ± 5.17^b^	26.38 ± 4.04^ab^	31.49 ± 4.95^b^
Year 2	−18.47 ± 4.64^ab^	−19.47 ± 5.39^ab^	−13.90 ± 4.87^ab^	−17.10 ± 5.51^ab^	−31.66 ± 4.04^a^	−14.79 ± 4.14^b^
Δ Body condition						
Year 1	−0.11 ± 0.07^a^	−0.14 ± 0.08^a^	−0.11 ± 0.09^a^	0.11 ± 0.09^a^	0.05 ± 0.07^a^	−0.15 ± 0.08^a^
Year 2	−0.11 ± 0.08^a^	0.06 ± 0.09^ab^	0.22 ± 0.08^b^	0.22 ± 0.09^ab^	0.12 ± 0.7^ab^	0.15 ± 0.7^ab^

Means within rows lacking common superscript differ (*P* < 0.05).

## DISCUSSION

Our research suggests that year and cow age have greater impacts on beef cattle performance, supplement intake, and grazing behavior than post-weaning heifer RFI in a winter grazing environment. However, differences in years observed for supplement intake variables are probably related to differences in supplement formulation as weather and forage conditions were similar both years of the study during the time period when supplement intake behavior was measured (Wyffels et al., 2020). Bovibox HM, which was used during year 1, contains 1.5% more magnesium oxide than Bovibox, which was used during year 2. The increase in magnesium oxide can increase bitterness and decrease palatability and probably altered animal supplement intake behavior.

Previous research has reported that low RFI cattle grazing summer rangelands in central Idaho travel further and graze longer than high RFI cattle (Sprinkle et al., 2019). The authors attributed this to high RFI cattle having greater heat of fermentation and as a result less tolerant of high temperatures. In contrast, we observed no effect of RFI on winter grazing beef cattle behavior. Temperatures were substantially cooler than what was reported by Sprinkle et al. (2019). Previous research has reported that while grazing late season dormant rangelands in Idaho that low RFI 2 yr olds lost less weight and body condition compared with high RFI 2 yr olds with no difference in daily distance traveled or foraging rate (bites ∙ m^−1^; Sprinkle et al., 2020). Conversely, we observed no effect of RFI on body weight or body condition change, and the differences we observed in distance traveled were associated with age rather than RFI classification. Our results are consistent with Meyer et al. (2008), where RFI did not affect body weight and condition change or supplement intake while grazing late winter and early spring. Thus, post-weaning RFI may be independent of mature cow body weight and have little impact on cow productivity and use of dormant forage (Walker et al., 2015). However, we believe that further research is needed to investigate the relationship of heifer post-weaning RFI classification on landscape use patterns, and foraging behavior, as well as the relationship between heifer post-weaning RFI classification and dry matter intake at different cow ages and stages of production.
